# Ultrasound shear wave elastography for assessing diaphragm function in mechanically ventilated patients: a breath-by-breath analysis

**DOI:** 10.1186/s13054-020-03338-y

**Published:** 2020-11-27

**Authors:** Quentin Fossé, Thomas Poulard, Marie-Cécile Niérat, Sara Virolle, Elise Morawiec, Jean-Yves Hogrel, Thomas Similowski, Alexandre Demoule, Jean-Luc Gennisson, Damien Bachasson, Martin Dres

**Affiliations:** 1 Sorbonne Université, INSERM, UMRS1158 Neurophysiologie respiratoire expérimentale et clinique, Paris, France; 2 AP-HP. Sorbonne Université, Hôpital Pitié-Salpêtrière, Service de Pneumologie, Médecine intensive – Réanimation (Département “R3S”), 75013 Paris, France; 3 Institut de Myologie, Laboratoire de Physiologie et d’Evaluation Neuromusculaire, Paris, France; 4 Laboratoire d’Imagerie Biomédicale Multimodale, BioMaps, Université Paris-Saclay, CEA, CNRS UMR 9011, INSERM UMR1281, SHFJ, Orsay, France

**Keywords:** Diaphragm, Diaphragm dysfunction, Mechanical ventilation, Ultrasound imaging, Shear wave elastography, Transdiaphragmatic pressure, Intensive care unit

## Abstract

**Background:**

Diaphragm dysfunction is highly prevalent in mechanically ventilated patients. Recent work showed that changes in diaphragm shear modulus (ΔSMdi) assessed using ultrasound shear wave elastography (SWE) are strongly related to changes in Pdi (ΔPdi) in healthy subjects. The aims of this study were to investigate the relationship between ΔSMdi and ΔPdi in mechanically ventilated patients, and whether ΔSMdi is responsive to change in respiratory load when varying the ventilator settings.

**Methods:**

A prospective, monocentric study was conducted in a 15-bed ICU. Patients were included if they met the readiness-to-wean criteria. Pdi was continuously monitored using a double-balloon feeding catheter orally introduced. The zone of apposition of the right hemidiaphragm was imaged using a linear transducer (SL10-2, Aixplorer, Supersonic Imagine, France). Ultrasound recordings were performed under various pressure support settings and during a spontaneous breathing trial (SBT). A breath-by-breath analysis was performed, allowing the direct comparison between ΔPdi and ΔSMdi. Pearson’s correlation coefficients (*r*) were used to investigate within-individual relationships between variables, and repeated measure correlations (*R*) were used for determining overall relationships between variables. Linear mixed models were used to compare breathing indices across the conditions of ventilation.

**Results:**

Thirty patients were included and 930 respiratory cycles were analyzed. Twenty-five were considered for the analysis. A significant correlation was found between ΔPdi and ΔSMdi (*R* = 0.45, 95% CIs [0.35 0.54], *p* < 0.001). Individual correlation displays a significant correlation in 8 patients out of 25 (*r* = 0.55–0.86, all *p* < 0.05, versus *r* = − 0.43–0.52, all *p* > 0.06). Changing the condition of ventilation similarly affected ΔPdi and ΔSMdi. Patients in which ΔPdi–ΔSMdi correlation was non-significant had a faster respiratory rate as compared to that of patient with a significant ΔPdi–ΔSMdi relationship (median (Q1–Q3), 25 (18–33) vs. 21 (15–26) breaths.min^−1^, respectively).

**Conclusions:**

We demonstrate that ultrasound SWE may be a promising surrogate to Pdi in mechanically ventilated patients. Respiratory rate appears to negatively impact SMdi measurement. Technological developments are needed to generalize this method in tachypneic patients.

***Trial registration*:**

NCT03832231.

## Background

**Fig. 1:**
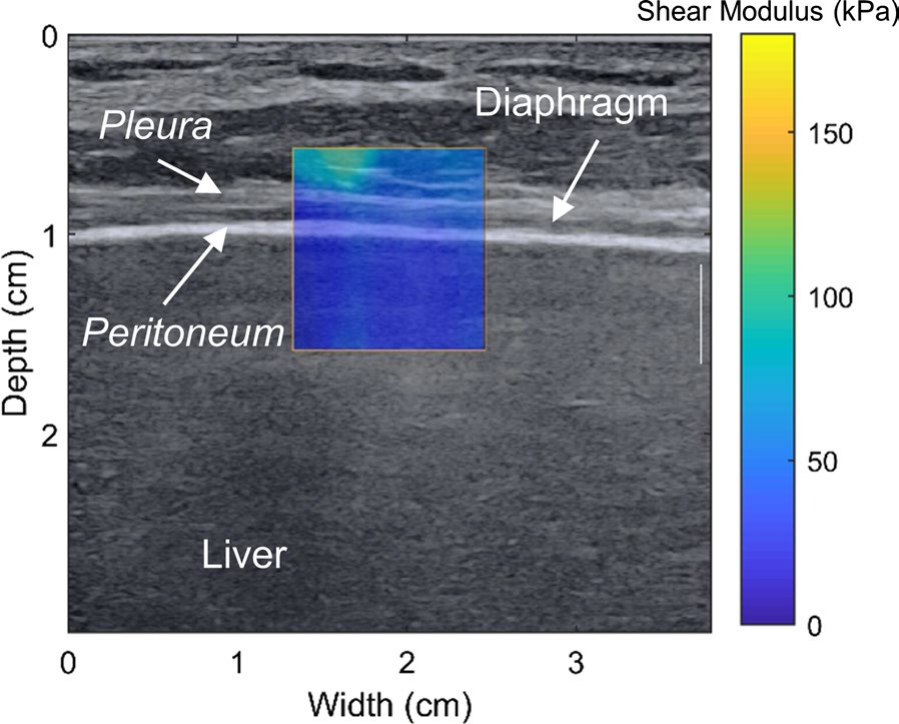
Typical ultrasound image obtained during shear wave elastography imaging of the diaphragm. Shear modulus map obtained from ultrasound shear wave elastography overlaid with standard ultrasound B-Mode during intercostal scanning of the diaphragm at the right zone of apposition

Acute respiratory failure is a common cause of admission in the intensive care unit (ICU) that can require invasive mechanical ventilation to relieve respiratory muscles work of breathing and ensure satisfactory gas exchange [1]. However, mechanical ventilation may produce harmful effects leading to the worsening of the patient’s prognosis independently of the primary reason for intubation [2]. More specifically, ventilator-induced diaphragm unloading results in time-dependent disuse atrophy of diaphragm myofibers [3, 4] and decreased diaphragm pressure-generating capacity [5, 6]. This is a serious issue that has been associated with prolonged duration of mechanical ventilation, difficult and prolonged weaning, and increased mortality [7–11]. Minimizing the ventilator unloading-induced diaphragm dysfunction may become a critical goal in the management of mechanically ventilated patients [12]. This strategy, namely the diaphragm protective ventilation [13], could not be implemented without a reliable and easy accessible monitoring of the diaphragm function. However, monitoring the diaphragm function in the ICU is not straightforward [7] as the gold standard relies on the recording of transdiaphragmatic pressure (Pdi) that is not widely available [14]. The later explains the growing interest of diaphragm ultrasound (US) as it provides direct visualization of muscle structure and functioning [15]. Diaphragm US is an interesting tool for assessing diaphragm function [16], monitoring its temporal structure changes (thickness, thickening, excursion, [3, 17]) and predicting weaning outcome [9, 16–19]. Beyond standard US imaging, ultrasound shear wave elastography (SWE) allows direct and real-time quantification of the mechanical properties of tissues [20]. Briefly, SWE relies on the measurement of the propagation velocity of shear waves remotely generated inside tissues by ultrasonic focused beams. This measurement is performed in three main steps. First an US pushing beam is focused remotely within the tissue. This results in the propagation of transient shear waves, propagating parallel to the surface of the US probe. Second, the probe switches to an ultrafast imaging mode, allowing the tracking of the propagating shear wave along the imaging plane. Finally, after estimating the shear wave speed between two points of the image, the US scanner is able to reconstruct a shear modulus (i.e. stiffness) map of the region of interest [20]. A typical B-Mode image overlaid with the elastography map is presented in Fig. 1. Local muscle shear modulus (i.e. stiffness) measured using SWE has been shown to provide reliable estimates of passive and active muscle force in locomotor muscles [21, 22]. Lately, we demonstrated that changes in diaphragm shear modulus (SMdi) reflect changes in Pdi during isovolumetric inspiratory efforts and ventilation against inspiratory loading in healthy subjects [23]. Recently, Flatres et al. performed measurements of shear modulus in the diaphragm (i.e. at the end of expiration only) and in limb muscles of critically ill patients and found a good intra- and inter-operator reliability, but the relationship with diaphragm function was not investigated [24]. Therefore, the present study aimed at investigating: (i) the agreement between changes in SMdi and changes in transdiaphragmatic pressure in mechanically ventilated patients and (ii), whether or not changes in SMdi are responsive to respiratory load when varying the ventilator settings. Secondary objective was to investigate changes in SMdi in patients undergoing a spontaneous breathing trial.

## Methods

This study followed the STROBE guidelines for observational study [25]. It was conducted in a medical 15-bed ICU from February 2019 to February 2020. It was approved by an ethical committee (ID RCB: 2018-A022311-54) and referenced on ClinicalTrials.gov (NCT03832231). Written informed consent was obtained from all patients or their relatives.

### Participants

Patients older than 18 years old were eligible for inclusion if they had been intubated and ventilated for at least 24 h, and failed a first spontaneous breathing trial (SBT). They could be included if they met predefined readiness-to-wean criteria on daily screening [26] and were therefore ready for a second SBT. Readiness-to-wean criteria were the following: SaO_2_ > 90% or PaO_2_/FiO_2_ ≥ 150 mmHg with a fraction of inspired oxygen (FiO_2_) ≤ 40%, no or minimal vasopressor, and a positive end-expiratory pressure (PEEP) ≤ 8 cmH_2_O. Patients who were pregnant, under a legal protection measure, with a contraindication to the insertion of a gastric-esophageal probe (esophageal bleeding), or with known allergies to anesthetizing were not included.

### Flow and pressure measurements

A flow sensor (Hamilton Medical, Bonaduz, Switzerland) connected to a spirometer (ADInstruments, Bella Vista, Australia) was used to continuously measure flow. Esophageal (Pes) and gastric (Pga) pressures were monitored using a double-balloon feeding catheter (NutriVentTM, Mirandola, Modena, Italy). The catheter was inserted through the mouth or nostril in the esophagus as demonstrated by the appearance of cardiac artifacts and appropriate negative swings of pressure during inspiration. Both balloons were inflated with 3 to 4 ml of air and connected to separated differential pressure transducers (model DP45-32, Validyne, Northridge, CA). The correct position of the esophageal balloon was confirmed by a dynamic occlusion test allowing the visualization of a corresponding deflation in esophageal pressure and airway pressure during inspiratory effort [27]. Flow and pressure signals were digitized (Powerlab, ADInstruments, Sydney, Australia) and recorded at a sampling frequency of 1 kHz (LabChart, ADInstruments). Pdi was obtained by the online subtraction of Pes from Pga.

### Ultrasound imaging and shear wave elastography

The zone of apposition of the right hemidiaphragm was imaged using an ultrafast US scanner (Aixplorer, Supersonic Imagine, France) driving a linear transducer array (SL 10-2, Supersonic Imagine). The probe was placed on the mid-axillary line, vertical to the chest wall, at the 8th–11th intercostal space and the spot was skin marked. US gel was generously applied to optimize acoustic coupling and minimal pressure was applied to the transducer to limit tissue deformation and/or alteration of breathing mechanics. In this location, the diaphragm appears as a three-layered structure just superficial to the liver, consisting of a relatively non-echogenic muscular layer bounded by two echogenic lines corresponding to the diaphragm *pleura* and *peritoneum* (Fig. 1). The rotation and angle of the transducer were then finely adjusted to obtain maximal echo intensity from diaphragm *pleura* and *peritoneum*. Using the built-in SWE mode of the US scanner, the region of interest was placed at the desired depth to fully cover the diaphragm. The sampling rate of SWE ranged from 1.6 to 2 Hz, depending on diaphragm depth. B-mode images were simultaneously displayed on the US scanner with a frame rate of 12 Hz. B-mode frames and shear wave velocity modulus values maps were retrieved from the US scanner for off-line processing. All US measurements were taken by a single operator (QF).

### Study protocol

Patients were in a semi-recumbent position throughout the study. Sedations were not modified during the protocol. The study was carried out as follows: i) recordings under different conditions of pressure support ventilation, ii) recordings during a SBT.

#### Conditions of mechanical ventilation

At baseline, patients were ventilated under pressure support ventilation mode. In each patient, 4 consecutive conditions of ventilation were applied in a randomized order: (i) initial ventilator settings predefined by the attending physician (PS), (ii) + 25% pressure support with baseline PEEP (PS_+25%_), (iii) -25% pressure support with baseline PEEP (PS_-25%_) and (iv) baseline pressure support and zero end-expiratory pressure (PS_ZEEP_). Each breathing condition was maintained for 10 min with 30-s acquisitions performed at 3 and 9 min within the condition.

#### Spontaneous breathing trial

Every patient underwent a planned 30 min SBT during which no assistance was provided from the ventilator (pressure support and PEEP were set at 0 cmH_2_O). This modality of SBT, part of usual care of our ICU, reflects the work of breathing after extubation [28]. Thirty seconds US and pressure recordings were performed at the onset of the SBT and every five minutes, for a maximum of 30 min. Failure of the SBT was defined by the following criteria: respiratory rate ≥ 35 breaths/min or increase ≥ 50%, SpO2 ≤ 90% or PaO2 ≤ 50 mmHg (with FiO2 ≥ 50%), heart rate ≥ 140 beats/min, new onset of supraventricular or ventricular arrhythmia, systolic arterial pressure > 180 or < 90 mmHg, alteration of consciousness, and diaphoresis or any signs of respiratory distress [26]. In case of failure of the SBT, initial ventilator settings were resumed. Otherwise, the SBT was defined as successful and the decision of extubation was taken by the clinician in charge.

### Data analysis

#### Analysis of shear modulus maps

Data were analyzed offline using standardized MATLAB (Mathworks, Natick, MA, USA) scripts. A rectangular region of interest (ROI) was drawn manually in the center of the diaphragm on the first frame of each recording. The ROI was replicated on subsequent frames. Diaphragm shear modulus was calculated as SMdi = ρ ⋅Vs^2^, where Vs is the velocity of the shear wave and ρ is the muscle density (1000 kg/m^3^). SMdi was reported as the median shear modulus within each ROI.

#### Breath-by-breath analysis

Changes in Pdi (ΔPdi), Pes (ΔPes), Pga (ΔPga) and SMdi (ΔSMdi) were computed for each respiratory cycle. The cycles were delimited by the deflations of the esophageal pressure signal and not with the flow signal since it could mask the onset of the inspiratory effort, especially when the patient has to overcome intrinsic PEEP [29, 30]. During inspiration, ΔPes was computed as the difference between the start of the decrease in Pes and the negative peak value of Pes. ΔPga was computed as the difference between the start of the increase in Pga and the positive peak value of Pga during inspiration. ΔPdi was computed as the difference between the start of the increase in Pdi and the positive peak value of Pdi during inspiration. ΔSMdi was calculated as the difference between the value of SMdi at the start of inspiration the positive peak value of SMdi during inspiration. Transdiaphragmatic pressure time product (PTPdi) per breath was also computed [31]. For every ventilatory condition, the 3 cycles with the least variation in ΔPdi were considered as representative of a given ventilatory condition and selected for further analysis. Maximal transdiaphragmatic pressure (Pdi_max_) was measured before the SBT using a one-way valve allowing exhalation only [32]. Pdi_max_ was calculated as the difference between Pdi at functional residual capacity and maximal Pdi during the Mueller maneuver. A video showing US imaging along with the temporal evolution of flow, internal pressures, and SMdi is available in Additional file 1: S1.

### Statistics

Results are presented as median (Q1-Q3) for descriptive statistics. We calculated our sample size based on the expected correlation between ΔPdi and ΔSMdi of 0.7 [23]. A required sample size of 17 patients was obtained. Sample size was then increased to compensate for any patients that would have to be withdrawn from the study for any reason. Differences in measured variables across the conditions of ventilation were assessed using linear mixed models. Linear mixed models were chosen over traditional two-way repeated ANOVAs given their ability to handle unbalanced data [33]. More specifically, six patients who failed the SBT had only one measurement during the SBT so that they would have been excluded from analysis using two-way repeated ANOVAs. Linear mixed models were also used to compare measured variables between patients based on the outcome of the SBT (success or failure) and to test the interaction effect between ventilatory condition × SBT outcome. Data from the ninth minute of each PS conditions and during the first and last measurements of the SBT were used for linear mixed models. Tukey’s post hoc tests were performed when a significant main or interaction effect was found.

Pearson’s correlation coefficients were used to investigate within-individual relationships between variables. Repeated measure correlation (R, 95% CIs) was used for determining overall relationships between variables [34]. Paired t-tests were used to compare differences in breathing pattern, diaphragm function, and SMdi between the SBT start and end-points for the failure and success groups. Unpaired t-tests were used to compare differences in breathing pattern, diaphragm function, and SMdi between patients who failed and succeeded the SBT. Unpaired t-tests were also used to compare differences in patients’ characteristics (age and BMI), breathing pattern, diaphragm function, and ΔSMdi between patients who presented a significant (*p* < 0.05) ΔPdi–ΔSMdi correlation and their counterparts. Analyses were performed in the computing environment R [35]. Significance was set at *p* < 0.05 for all tests.

## Results

### Population

**Fig. 2:**
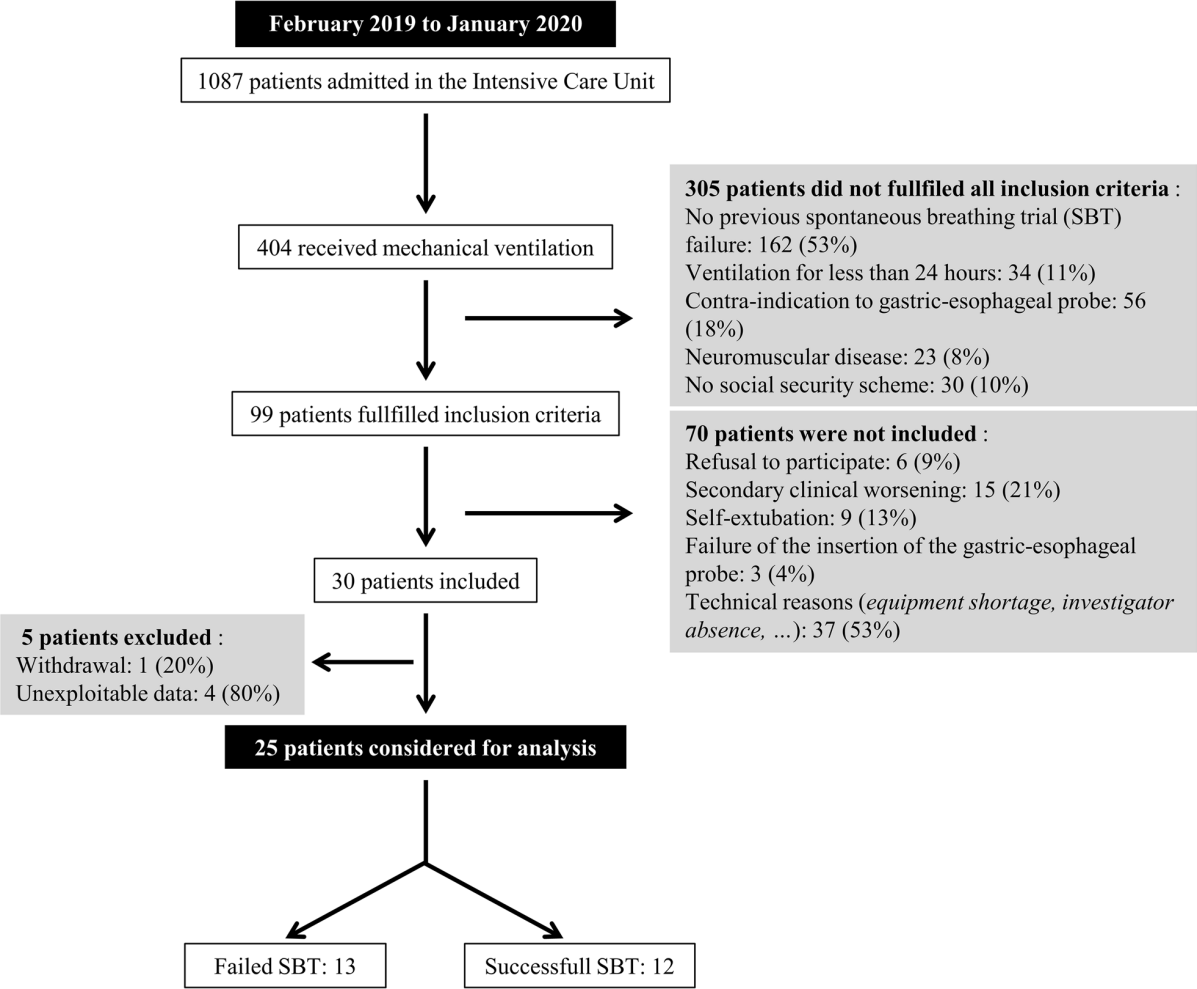
Flowchart of the study

Between February 2019 and February 2020, 1087 patients were admitted within the ICU. 404 patients received invasive mechanical ventilation, 99 fulfilled the inclusion criteria leading to the enrollment of 30 patients. Twenty-five patients were considered for subsequent analysis (see the flow chart of the study in Fig. 2). The main characteristics of patients are displayed in Table 1. The main reason for intubation was acute respiratory failure, and patients were ventilated since 4 (3–7) days at the time of inclusion. At baseline, patients were receiving a pressure support level of 10 (10–13) cmH_2_O and a PEEP level of 5 (5–5) cmH_2_O. A total of 3878 breathing cycles were recorded and 930 were considered for further analysis (i.e. corresponding to triplicate for each condition of ventilation). All patients tolerated each condition of the protocol.

**Table 1:** Characteristics of patients at inclusion

Characteristics	Values
*Demographics*	
Number	25
Age, years	65 (58–75)
Female, *n* (%)	6 (24%)
Body mass index, kg·m^−2^	25 (22–28)
*Previous conditions*	
COPD, *n* (%)	9 (38%)
Chronic cardiac disease, *n* (%)	12 (50%)
Chronic kidney disease, *n* (%)	5 (21%)
Current smoking, *n* (%)	6 (25%)
*ICU stay descriptors*	
SAPS 2 score	51 (39–62)
SOFA score	5 (4–8)
Duration of intubation, days	4 (3–7)
*Main raison for intubation*	
Hypoxemic acute respiratory failure, *n* (%)	10 (40%)
Coma, *n* (%)	10 (40%)
Hypercapnic acute respiratory failure, *n* (%)	3 (12%)
Cardiac arrest, *n* (%)	2 (8%)
*Ventilator settings*	
Pressure support, cmH2O	10 (10–12)
PEEP, cmH2O	5 (5–5)
FiO_2_, %	30 (30–40)
*Arterial blood gases*	
pH	7.4 (7.38–7.49)
PaO_2_/FiO_2_	273 (170–312)
PaCO_2_, mmHg	49 (40–58)
Maximal inspiratory pressure, cmH_2_O	24 (17–35)
Number of spontaneous breathing trial, *n*	1 (1–2)

Results are shown as number (%) or median (Q1–Q3). SAPS II, simplified acute physiology score; SOFA, Sequential organ failure assessment. PEEP, positive end-expiratory pressure

### Changes in diaphragm shear modulus and diaphragm function across breathing conditions

**Fig. 3:**
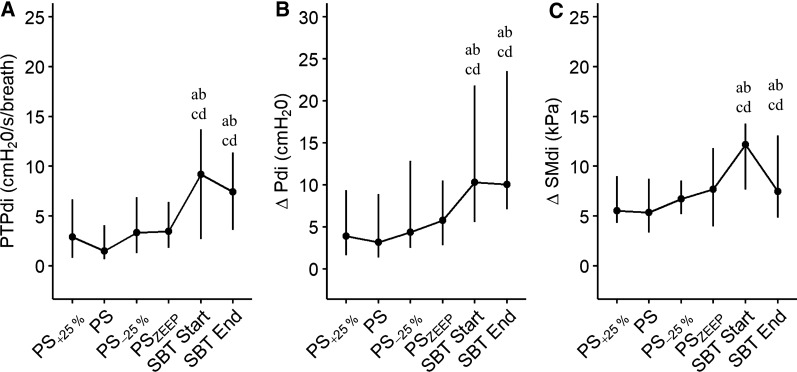
Diaphragm shear modulus and transdiaphragmatic pressure across different breathing conditions. PTPdi, pressure–time product of transdiaphragmatic pressure (Panel **a**); ΔPdi, inspiratory change in transdiaphragmatic pressure (Panel **b**); ΔSMdi, inspiratory change in diaphragm shear modulus assessed using ultrasound shear wave elastography (Panel **c**). The error bars correspond to 25th and 75th percentile. PS, pressure support ventilation with baseline inspiratory support and positive end-expiratory pressure; PS_+25%_, PS with inspiratory pressure support increased by 25%; PS_-25%_, PS with inspiratory pressure support decreased by 25%; PS_ZEEP_, PS with baseline inspiratory support and positive end-expiratory pressure set at 0; SBT, spontaneous breathing trial; SBT Start, start of the SBT; SBT End, end of the SBT. **a** Significantly different from PS_+25%_; **b** Significantly different from PS; **c** significantly different from PS_−25%_; **d **Significantly different from PS_ZEEP_ (all* p* < 0.05)

Changes in breathing pattern and ΔPes, ΔPga, ΔPdi, PTPdi, and ΔSMdi at each condition of the protocol are shown in Table 2, and time course of ΔPdi, PTPdi, and ΔSMdi during the protocol is displayed in Fig. 3. There was a significant relationship between the level of ventilatory assistance and the breathing pattern, namely the respiratory rate increased and the tidal volume decreased while the ventilatory assistance decreased. Similarly, both PTPdi and ΔPdi significantly increased while the level of assistance decreased. Under PS ventilatory conditions, ΔPdi ranged between 0.1 and 38.1 cmH_2_O and between 0.6 and 50.7 cmH_2_O during the SBT. Similarly, ΔSMdi presented a stepwise increase corresponding to each decrease of the ventilatory assistance level.

**Table 2:** Physiological variables and diaphragm shear modulus under the different ventilatory conditions

Variables	Condition of ventilation					
	PS_+25%_	PS	PS_-25%_	PS_ZEEP_	SBT Start	SBT End
Pressure support, cmH_2_O	13 (12–16)	10 (10–13)	8 (7–9)	10 (10–13)	0	0
PEEP, cmH_2_O	5 (5–5)	5 (5–5)	5 (5–5)	0	0	0
V_T_, mL/kg	5.7 (4.9–7.4)^def^	4.9 (4.2–6.2)^ef^	4.6 (4.1–5.4)^ace^	4.8 (3.8–6.8)^ae^	3.3 (3.1–4.9)^abcd^	3.9 (3.2–5.4)^ab^
Respiratory rate, cycles/min	19 (13–25)	21 (17–27)	23 (18–29)	24 (17–30)	24 (18–33)^ab^	26 (22–32)^a^
Inspiratory time, s	1.1 (1.0–1.6)	1.0 (0.9–1.3)	1.0 (0.9–1.2)^a^	1.0 (0.8–1.3)	1.2 (0.9–1.3)^c^	1.1 (0.9–1.3)^a^
Expiratory time, s	2.0 (1.5–2.8)	1.8 (1.3–2.4)	1.5 (1.2–2.1)	1.5 (1.2–2.2)	1.3 (1.0–1.8)^ab^	1.3 (1.0–1.6)
Inspiratory duty cycle	0.40 (0.37–0.45)^e^	0.41 (0.36–0.45)	0.39 (0.37–0.43)^e^	0.41 (0.38–0.43)^e^	0.45 (0.43–0.50)^acd^	0.45 (0.41–0.47)
Minute ventilation (l.min^−1^)	7.8 (5.8–10.4)	8.1 (6.0–10.1)	7.9 (6.1–9.5)	8.2 (5.8–9.4)	6.4 (5.2–9.0)	7.5 (5.5–8.6)
ΔPdi, cmH_2_O	3.9 (1.6–9.4)^ef^	3.2 (1.3–8.9)^ef^	4.3 (2.5–12.9)^ef^	5.8 (2.8–10.5)^ef^	10.3 (5.6–21.8)^abcd^	10.0 (7.1–23.5)^abcd^
PTPdi, cmH_2_O.s/breath	2.9 (0.8–6.7)^ef^	1.5 (0.7–4.1)^ef^	3.4 (1.3–6.9)^ef^	3.5 (1.8–6.4)^ef^	9.2 (2.7–13.7)^abcd^	7.4 (3.6–11.4)^abcd^
PTPdi, cmH_2_O.s/min	42 (17–131)^ef^	33 (16–137)^ef^	78 (23–128)^ef^	78 (34–167)^f^	188 (66–369)^abc^	178 (103–297)^abcd^
ΔPes, cmH_2_O	− 1.7 (− 8.5 to − 0.9)^ef^	− 1.7 (− 9.0 to − 0.9)^ef^	− 2.9 (− 12.2 to − 1.9)^ef^	− 4.8 (− 8.8 to − 2.8)^ef^	− 11.3 (− 18.7 to − 8.6)^abcd^	− 10.6 (− 23.5 to − 9.3)^abcd^
ΔPga, cmH_2_O	0.9 (0.2–2.3)	1.1 (0.2–1.9)	1.0 (0.5–2.3)	0.9 (0.3–2.4)	0.4 (0.0–1.3)	0.2 (0.0–1.0)
ΔSMdi, kPa	5.5 (3.8–9.0)^ef^	5.4 (3.5–8.8)^ef^	7.0 (5.8–8.6)^ef^	7.7 (4.0–11.8)^ef^	12.2 (7.7–14.3)^abcd^	7.5 (4.8–13.1)^abcd^

Results are shown as median (Q1–Q3). PEEP, positive end-expiratory pressure; V_T_, tidal volume; ΔPdi, inspiratory change in transdiaphragmatic pressure; PTPdi, pressure–time product of Pdi; ΔPes, inspiratory changes in esophageal pressure; ΔPga, inspiratory changes in gastric pressure; ΔSMdi, inspiratory change in diaphragm shear modulus assessed using ultrasound shear wave elastography; PS, pressure support ventilation with baseline inspiratory support and positive end-expiratory pressure; PS_+25%_, PS with inspiratory pressure support increased by 25%; PS_−25%_, PS with inspiratory pressure support decreased by 25%; PS_ZEEP_, PS with baseline inspiratory support and zero end-expiratory pressure; SBT, spontaneous breathing trial. a, significantly different from PS_+25%_; b, Significantly different from PS; c, significantly different from PS_−25%_; d, significantly different from PS_ZEEP_; e, significantly different from the start of the SBT; f, significantly different from the end of the SBT; all *p* < 0.05

### Correlations between changes in diaphragm shear modulus and diaphragm function

**Fig. 4:**
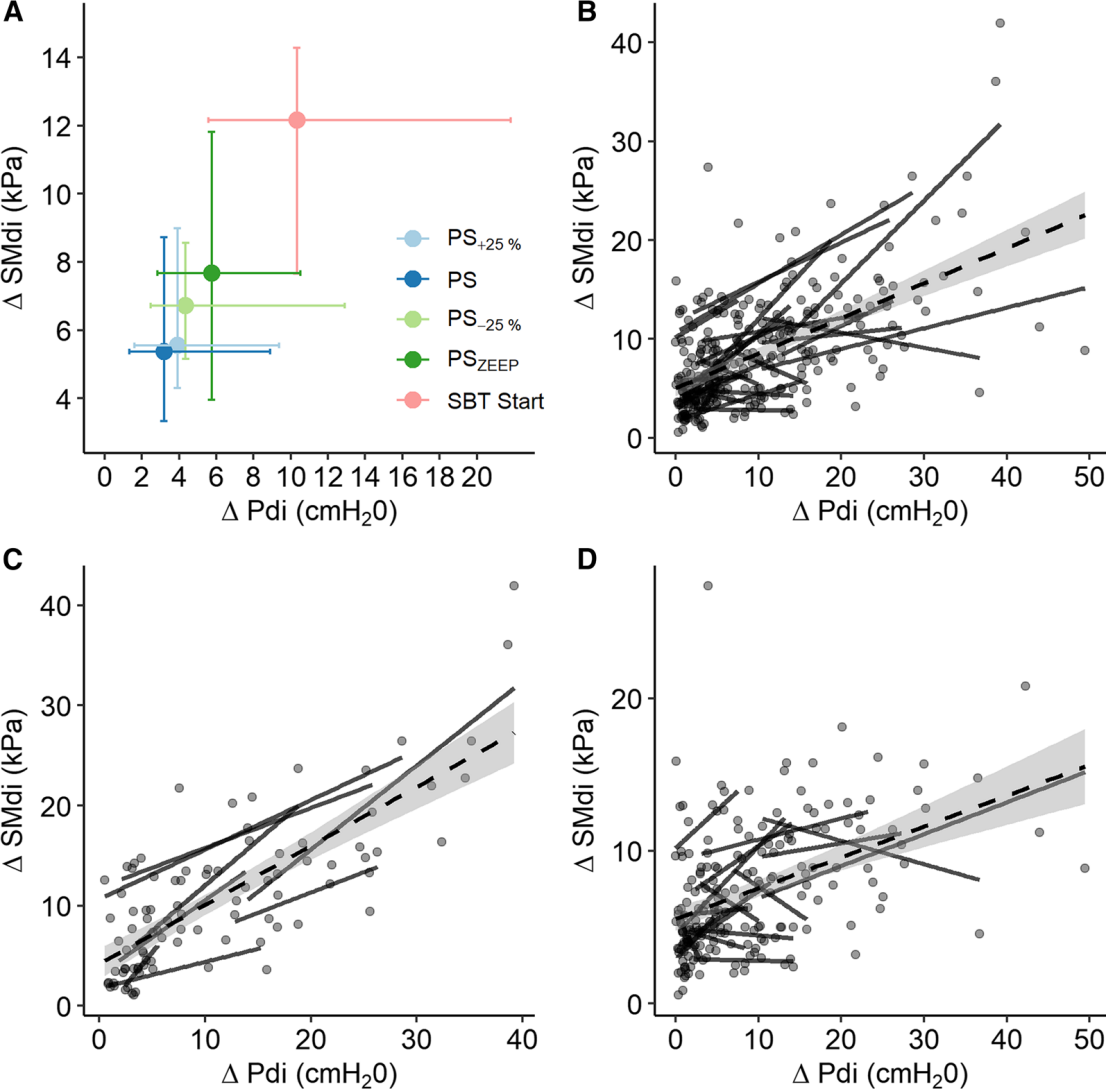
Relationship between changes in diaphragm shear modulus and changes in transdiaphragmatic pressure. Averaged data (panel **a**, data are shown as median (Q1–Q3)) and all data points with individual and overall linear regression lines (panel **b**). Panel** c** displays the individual linear regressions in patients with a significant ΔPdi-ΔSMdi correlation (*p* < 0.05). Panel** d** displays the individual linear regressions in patients with no significant ΔPdi-ΔSMdi correlation (*p* > 0.05). ΔPdi, inspiratory change in transdiaphragmatic pressure; ΔSMdi, inspiratory change in diaphragm shear modulus assessed using ultrasound shear wave elastography; PS, pressure support ventilation with baseline inspiratory support and positive end-expiratory pressure; PS_+25%_, PS with inspiratory pressure support increased by 25%; PS_−25%_, PS with inspiratory pressure support decreased by 25%; PS_ZEEP_, PS with baseline inspiratory support and positive end-expiratory pressure set at 0; SBT Start, start of the spontaneous breathing trial. In panel **a**., only cycles gathered at the end of each condition and at the start of SBT were used. In panel **b**, cycles gathered at all time-points were used

**Fig. 5:**
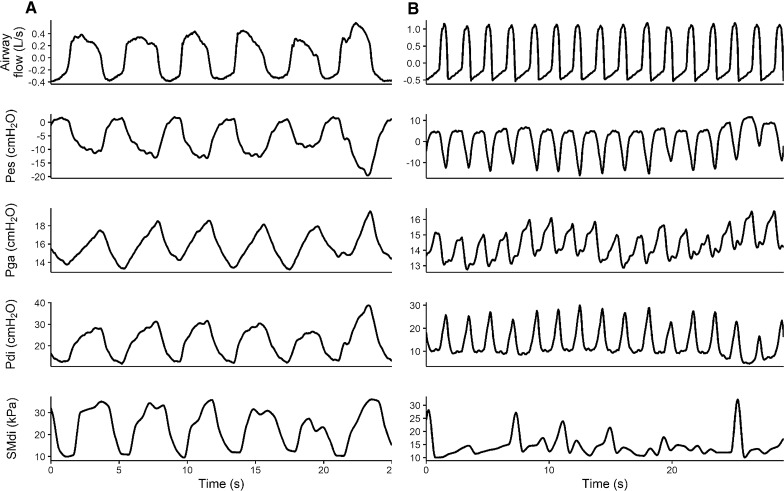
Physiological variables and diaphragm shear modulus over time in two patients. Temporal evolution of airway flow, esophageal (Pes), gastric (Pga) and transdiaphragmatic (Pdi) pressures, and diaphragm shear modulus (SMdi) in a patient with a significant ΔPdi–ΔSMdi relationship (r = 0.81, *p* = 0.002, panel **a**) and in a patient with a non-significant ΔPdi–ΔSMdi relationship (r = 0.14, p = 0.643, panel **b**). Respiratory rates were of 12 and 33 breaths.min^−1^ for panel **a** and **b**, respectively

Figure 4 shows individual and global correlations between ΔSMdi and ΔPdi. Repeated measure correlation showed a significant overall correlation between ΔSMdi and ΔPdi (*R* = 0.45, 95% CIs [0.35 0.54], *p* < 0.001). Regarding within-subject correlation analysis, ΔSMdi and ΔPdi exhibited a significant correlation in 8 patients and no significant correlation in 17 patients (see Supplemental Information S2 for individual correlation coefficient and *p* values). Figure 5 shows the temporal evolution of the airway flow, Pes, Pga, Pdi and SMdi in a patient with a strong ΔPdi–ΔSMdi correlation (*r* = 0.81, *p* = 0.002) and in a patient with no ΔPdi–ΔSMdi correlation (*r* = 0.14, *p* = 0.643) during the protocol. Patients in which ΔPdi–ΔSMdi correlation was non-significant were older, had a faster respiratory rate, lower ΔPdi, lower PTPdi and lower ΔPga as compared to that of patients with significant ΔPdi–ΔSMdi correlation (Table 3). No difference in BMI was found between patients with and without a significant ΔPdi–ΔSMdi correlation (Table 3).

**Table 3:** Characteristics of patients, changes in breathing pattern, diaphragm function, and diaphragm shear modulus in patients with and without a significant correlation between changes in transdiaphragmatic pressure and changes in diaphragm shear modulus

	Significant ΔPdi-ΔSMdi correlation	Non-significant ΔPdi-ΔSMdi correlation	*p* value
	(*n* = 8)	(*n* = 16)	
*Patients’ characteristics*			
Age, years	62 (57 to 71)	73 (60 to 79)	< 0.001
Body mass index, kg·m^−2^	24.6 (18.0 to 27.0)	25.8 (23.2 to 30.4)	0.224
*Breathing pattern*			
V_T_, mL/kg	4.8 (3.9 to 6.0)	4.6 (3.8 to 6.1)	0.818
Respiratory rate, cycles/min	21 (15 to 26)	25 (18 to 33)	< 0.001
*Diaphragm function*			
ΔPdi, cmH_2_O	7.5 (3.3 to 16.9)	6.0 (1.8 to 13.2)	0.086
PTPdi, cmH_2_O.s/breath	4.5 (1.9 to 10.6)	4.3 (1.2 to 8.0)	0.019
PTPdi, cmH_2_O.s/min	91 (34 to 260)	102 (24 to 189)	0.088
ΔPes, cmH_2_O	−6.4 (−13.2 to −1.9)	−6.5 (−13.3 to −1.9)	0.443
ΔPga, cmH_2_O	1.5 (0.1–2.6)	0.6 (0.1–1.6)	0.004
ΔSMdi, kPa	9.4 (4.5–14.1)	6.3 (4.1–9.9)	< 0.001

Results are shown as median (Q1–Q3). *V*_*T*_, tidal volume; ΔPdi, inspiratory changes in transdiaphragmatic pressure; PTPdi, transdiaphragmatic pressure time product; ΔPes, inspiratory changes in esophageal pressure; ΔPga, inspiratory changes in gastric pressure; ΔSMdi, inspiratory changes in diaphragm shear modulus

### Comparison of diaphragm shear modulus in patients who failed and succeeded the spontaneous breathing trial

Thirteen patients (52%) failed the SBT. Reasons of failure were acute respiratory distress (5/13), neurologic impairment (4/13) and weaning induced pulmonary edema (4/13). ∆Pdi and PTPdi tended to be higher at the start of the SBT in patients who failed the SBT as compared to their counterpart. No difference was found regarding ∆SMdi between the two groups of patients. Table 4 presents the breathing pattern and clinical characteristics of patients at the start and the end of the SBT, based on the outcome of the SBT.

**Table 4:** Physiological variables and diaphragm shear modulus according to the outcome of the spontaneous breathing trial

	SBT success (*n* = 12)			SBT failure (*n* = 13)		
	Start of SBT	End of SBT	*p* value	Start of SBT	End of SBT	*p* value
*Clinical variables*						
Systolic AP, mmHg	140 (129–157)	126 (121–159)	0.109	136 (124–150)	158 (128–161)	0.045
Heat rate, min^−1^	93 (84–99)	97 (81–103)	0.541	110 (90–118)	111 (95–121)	0.391
*Breathing pattern*						
V_T_, mL/kg	4.1 (3.2–5.6)	4.1 (3.4–5.4)	0.894	3.2 (2.9–3.3)	3.5 (2.9–4.9)	0.423
Respiratory rate, cycles/min	24 (18–34)	25 (19–26)	0.332	24 (20–29)	32 (27–34)	0.288
*Diaphragm function*						
ΔPdi, cmH_2_O	5.9 (4.1–17.4)	9.5 (5.2–12.0)	0.777	20.7 (12.5–29.1)	15.3 (8.8–27.5)	0.570
PTPdi, cmH_2_O.s/breath	3.2 (1.9–10.3)	5.7 (3.2–11.9)	0.903	11.6 (9.3–15.0)	7.9 (5.0–10.7)	0.495
PTPdi, cmH_2_O.s/min	70 (47–364)	138 (73–209)	0.853	239 (187–446)	263 (134–316)	0.602
ΔSMdi, kPa	9.8 (7.8–13.4)	7.4 (4.8–9.7)	0.323	13.5 (8.8–15.9)	7.6 (5.6–14.6)	0.879

Results are shown as median (Q1-Q3). SBT, spontaneous breathing trial; AP, arterial pressure; *V*_*T*_, tidal volume; ΔPdi, inspiratory changes in transdiaphragmatic pressure; PTPdi, transdiaphragmatic pressure time product; ΔSMdi, inspiratory changes in diaphragm shear modulus

## Discussion

The present work provides new insights regarding the use of ultrasound shear wave elastography for the assessment of diaphragm function in patients under mechanical ventilation using a breath-by-breath analysis. First, we found that changes in diaphragm shear modulus and changes in transdiaphragmatic pressure were significantly correlated. However, when considering the relationship between diaphragm shear modulus and transdiaphragmatic pressure within individuals, the correlation was significant in only a third of patients and it was absent in the remaining patients. Second, we found no significant difference in diaphragm function and change in diaphragm shear modulus in response to changes in ventilatory conditions. Changes in transdiaphragmatic pressure and in diaphragm shear modulus significantly increased during the SBT. Third, we found no significant difference regarding SBT-induced changes in diaphragm shear modulus between patients who failed or succeeded the spontaneous breathing trial.

### Diaphragm shear modulus as a surrogate of transdiaphragmatic pressure in mechanically ventilated patients

Changes in SMdi have been demonstrated to be strongly related to changes in mouth pressure during isovolumetric inspiratory efforts in healthy subjects (*R*^2^ = 0.94 ± 0.05) [36]. In a recent work, our group demonstrated a strong relationship between ΔSMdi and ΔPdi during both isovolumetric inspiratory effort and inspiratory threshold loading in healthy subjects [23]. Our findings in turn demonstrate in mechanically ventilated patients the linear relationship between ΔSMdi and ΔPdi (Fig. 4a). However, in the current work, ΔSMdi significantly correlated to ΔPdi in only one third of patients (Fig. 4b–d). There are several potential explanations for these findings. First, the range of ΔPdi measured, all conditions of ventilation considered, was largely smaller than those induced by isovolumetric inspiratory effort or inspiratory threshold loading (both performed between 0 and 60% of maximal inspiratory pressure) in our previous study. Consequently, the range over which ΔPdi and ΔSMdi values were measured (0–120 cmH_2_0) was much wider than the one in the present study (0–50 cmH_2_O with 62% of ΔPdi < 10 cmH_2_O). Therefore, the moderate relationship between ΔSMdi and ΔPdi observed in our study may be partly explained by the relatively low, albeit more physiological, range over which diaphragm effort was analyzed. Our results support this hypothesis, as patients with a significant ΔPdi–ΔSMdi correlation displayed higher ΔPdi (Table 3). Second, the sampling rate of SWE (~ 2 Hz) is a critical factor for the monitoring of cyclic activity of a muscle such as the diaphragm. Indeed, the computation of ΔSMdi relies on the assumption that both the maximal and minimal value of SMdi during a breathing cycle are successfully recorded. Consequently, tachypnea emerges as a major issue when ΔSMdi needs to be determined. As the patient increases its respiratory rate, it becomes likely that minimal and/or maximal SMdi values are flawed because the low sampling rate of SWE does not ensure that the measurement is performed at the end of inspiration and expiration. More precisely, maximal and minimal SMdi values are expected to occur at the end of the inspiration and expiration, respectively. An increase in respiratory rate leads to a reduction of the inspiratory time. As the number of SMdi values acquired during a breathing cycle is limited (i.e. two SMdi values per second), the shortening of the inspiratory time limits the likelihood of measuring both the minimal and maximal SMdi values during a given respiratory cycle. Consequently, **∆**SMdi may be underestimated when SMdi is not recorded at the very end of inspiration and/or expiration, thus weakening the relationship between ∆SMdi and ∆Pdi. Our results also support this idea. When ΔPdi–ΔSMdi correlation was not significant, patients presented a significantly higher respiratory rate compared to their counterparts (Table 3). Also, ΔSMdi were significantly lower in patients with no ΔPdi–ΔSMdi correlation compared to patients with a significant ΔPdi–ΔSMdi correlation. This finding corroborates the idea that ΔSMdi is underestimated when tachypnea occurs. To illustrate this phenomenon, videos showing US imaging along with the temporal evolution of flow, internal pressures, and SMdi in one patient with a low respiratory rate and one patient with a high respiratory rate are available in Additional file 1: S1 and Additional file 3: S3, respectively. These findings emphasize the issue that changes in diaphragm stiffness cannot be captured when tachypnea occurs, a situation that is frequent in critically ill patients. Therefore, substantial technological developments aiming at increasing the frame rate of SWE when used in the diaphragm are required to make its use generalizable in all ICU patients. Currently, SWE relies on the measurement of propagating shear wave velocity at multiple laterally spaced points. Recent work showed that by reducing the number of lateral points over which the shear wave velocity is calculated, accurate estimates of the mechanical properties of a viscoelastic material can be obtained [37]. This promising technique could significantly reduce the computational time needed to obtain a shear modulus map and theoretically increase the sampling rate of SWE by four. Such frame rate could improve the accuracy of SMdi measurement in the case of tachypnea. Combining SWE with previously identified indices such as diaphragm excursion, thickening fraction [19, 38, 39], tissue Doppler imaging [40], and strain [41] might also improve the performance of diaphragm US for gauging diaphragm function. Between-day, intra- and inter-operator reliability of diaphragm SWE elastography was not assessed in the current work. This shall be investigated when technical limitations, in particular regarding the frame rate, will be resolved. In summary, the absence of significant ΔPdi–ΔSMdi correlation in two-third of the patients included may be explained by two main factors: i) the narrower range of ΔPdi values in mechanically ventilated patients (0–50 cmH_2_O), as compared to our previous work in healthy subjects (0–120 cmH_2_O, [22]) and ii) the higher inspiratory rate observed in individuals for no significant ΔPdi–ΔSMdi correlation was found.

### The sensitivity of diaphragm shear modulus for detecting modification in respiratory load

As expected, removing PS was associated with a significant increase in diaphragm function as assessed using ΔPdi and PTPdi as repeatedly observed [30, 42, 43]. However, when the inspiratory support level or PEEP was modified, we found no significant change in PTPdi and ΔPdi as compared to baseline PS settings (Fig. 3). This might be explained by the chosen ventilatory condition. More specifically, increasing or decreasing PS by 25% led to relatively small absolute changes in PS. Similarly, removing PEEP but maintaining initial PS settings did not lead to an increase in PTPdi or ΔPdi, pointing out that the chosen ventilatory conditions appear to be too close to each other to detect changes in diaphragm function. Interestingly and despite limitations of SWE mentioned above, we found that ΔSMdi also increased during the SBT and that differences with other breathing conditions were identical to those observed in PTPdi and ΔPdi. These findings highlight that an increase in diaphragm function may be detected by diaphragm SWE that is a promising track in the field of noninvasive diaphragm function in the ICU.

### Comparison of patients who failed or succeeded the spontaneous breathing trial

A secondary aim of the present study was to investigate differences in diaphragm function and diaphragm shear modulus according to the outcome of a SBT. As previously reported, we observed that patients who failed the SBT had larger (i.e. almost four times higher) ΔPdi and ΔPTPdi at the start of the SBT as compared to patients who succeeded (Table 4, [44]). Regarding ΔSMdi, no difference was observed between patients who succeeded or failed the SBT, whether at the start or the end of the SBT. Possibly, the relatively small sample size, further divided in two groups exacerbated the limitations of SWE mentioned above, limiting its use to discriminate patients according to the SBT outcome in the present work. Also, the outcome of the SBT depends on a large range of clinical parameters (i.e. desaturation, increased arterial pressure, etc.), which would primarily differentiate patients succeeding or failing the SBT before any difference could be observed on ΔSMdi.

### Strength and limitations

The current work is based on a breath-by-breath analysis. All US acquisitions were synchronized with the physiological parameters. This method allowed a direct comparison of diaphragm indices for a given breathing cycle. This strategy ensures an unbiased comparison of the various diaphragm function indices, as ΔPdi and ΔSMdi are compared for the exact same diaphragm contraction. All data were analyzed offline, using standardized scripts, by an operator blinded to the ventilatory condition. Conversely, this study has several limitations. As mentioned above, the relatively low sampling rate of SWE hinders its applicability in tachypneic patients. Our team is currently working on the development of specific US sequences that would allow a significant increase in SWE sampling rate. Such improvement is needed to accurately measure ΔSMdi in case of tachypnea. In this work, PS and PEEP were purposely changed to increase or decrease respiratory load. However, no differences in ΔPdi, PTPdi or ΔSMdi were found across the conditions of PS ventilation. Inspiratory load was only significantly increased during the SBT. This may be explained by the limited range of inspiratory effort, which did not induce significant changes in diaphragm function.

## Conclusions

Monitoring changes in diaphragm shear modulus as assessed using ultrasound shear wave elastography is promising as a noninvasive and specific approach to assess diaphragm function within the ICU. However, limitations of ultrasound shear wave elastography arise from its limited sampling rate when tachypnea occurs. Further technological and methodological developments are required to optimize the use of diaphragm shear wave elastography for the ICU.

## Supplementary information

**Additional file 1**. **Figure S1**: Movie clip of temporal changes in esophageal pressure, gastric pressure, transdiaphragmatic pressure, and diaphragm shear modulus during pressure support ventilation in one patient with a breathing frequency of 12 breaths/min in which a strong correlation (*r* = 0.81,* p* = 0.002) was found between changes in transdiaphragmatic pressure (ΔPdi) and changes in diaphragm shear modulus (ΔSMdi) can be found at the following link: https://figshare.com/s/f53dbea5b18c420a1490.

**Additional file 2**. ** Figure S2**: Individual correlation coefficients between changes in transdiaphragmatic pressure (ΔPdi) and changes in diaphragm shear modulus (SMdi) can be found at the following link: https://figshare.com/s/c0f06ddf34d5a784ccb5.

**Additional file 3**. **Figure S3**: Movie clip of temporal changes in esophageal pressure, gastric pressure, transdiaphragmatic pressure, and diaphragm shear modulus during pressure support ventilation in one patient with a breathing frequency of 33 breaths/min in which no correlation (*r* = 0.14,* p* = 0.643) was found between changes in transdiaphragmatic pressure (ΔPdi) and changes in diaphragm shear modulus (ΔSMdi) can be found at the following link: https://figshare.com/s/fb33c7701fb50c35c98d.

## Data Availability

The datasets used and/or analyzed during the current study are available from the corresponding author on reasonable request.
