# Supplementary figures and images for: Ultrasound shear wave elastography for assessing diaphragm function in mechanically ventilated patients: a breath-by-breath analysis
Source: Crit Care. 2020 Nov 27;24:669. doi: 10.1186/s13054-020-03338-y (PMC7695240; doi:10.1186/s13054-020-03338-y)

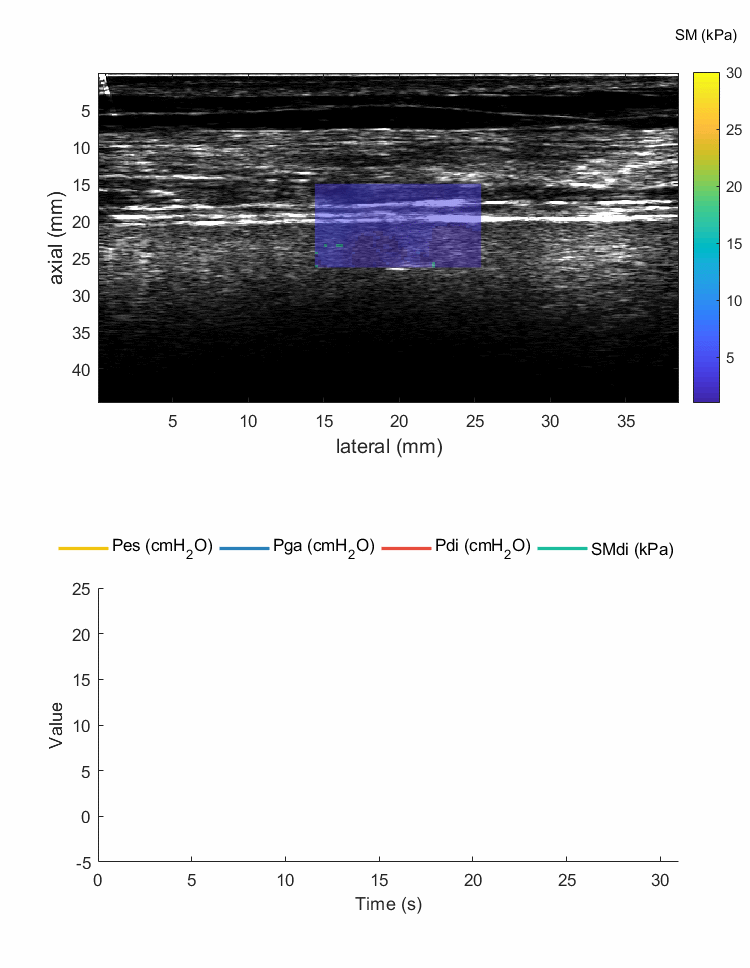

Supplement: Supplementary file 1 — Additional file 1. Figure S1: Movie clip of temporal changes in esophageal pressure, gastric pressure, transdiaphragmatic pressure, and diaphragm shear modulus during pressure support ventilation in one patient with a breathing frequency of 12 breaths/min in which a strong correlation (r = 0.81, p = 0.002) was found between changes in transdiaphragmatic pressure (ΔPdi) and changes in diaphragm shear modulus (ΔSMdi) can be found at the following link: https://figshare.com/s/f53dbea5b18c420a1490. [file 13054_2020_3338_MOESM1_ESM.gif]

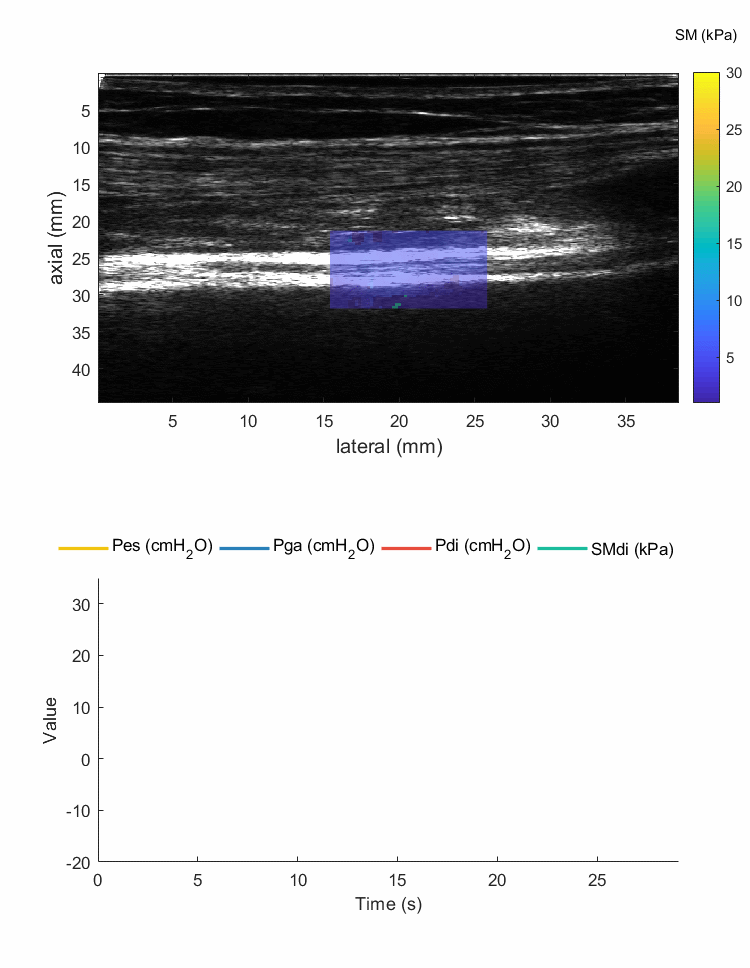

Supplement: Supplementary file 3 — Additional file 3. Figure S3: Movie clip of temporal changes in esophageal pressure, gastric pressure, transdiaphragmatic pressure, and diaphragm shear modulus during pressure support ventilation in one patient with a breathing frequency of 33 breaths/min in which no correlation (r = 0.14, p = 0.643) was found between changes in transdiaphragmatic pressure (ΔPdi) and changes in diaphragm shear modulus (ΔSMdi) can be found at the following link: https://figshare.com/s/fb33c7701fb50c35c98d. [file 13054_2020_3338_MOESM3_ESM.gif]
